# Optimising deep learning models for ophthalmological disorder classification

**DOI:** 10.1038/s41598-024-75867-3

**Published:** 2025-01-24

**Authors:** S. Vidivelli, P. Padmakumari, C. Parthiban, A. DharunBalaji, R. Manikandan, Amir H. Gandomi

**Affiliations:** 1 School of Computing, SASTRA University, Thanjavur, Tamilnadu India; 2 Department of CSE, SASTRA University, Thanjavur, Tamilnadu India; 3 Faculty of Engineering & Information Systems, University of Technology Sydney, Sydney, NSW 2007 Australia; 4 University Research and Innovation Center (EKIK), Óbuda University, Budapest, 1034 Hungary

**Keywords:** Transfer learning, DenseNet, ResNet, Lenet, Adam, Stochastic gradient descent, Computational science, Computer science

## Abstract

Fundus imaging, a technique for recording retinal structural components and anomalies, is essential for observing and identifying ophthalmological diseases. Disorders such as hypertension, glaucoma, and diabetic retinopathy are indicated by structural alterations in the optic disc, blood vessels, fovea, and macula. Patients frequently deal with various ophthalmological conditions in either one or both eyes. In this article, we have used different deep learning models for the categorisation of ophthalmological disorders into multiple classes and multiple labels utilising transfer learning-based convolutional neural network (CNN) methods. The Ocular Disease Intelligent Recognition (ODIR) database is used for experiments, and it contains fundus images of the patient’s left and right eyes. We compared the performance of two different optimisers, Stochastic Gradient Descent (SGD) and Adam, separately. The best result was achieved using the MobileNet model with the Adam optimiser, yielding a testing accuracy of 89.64%.

## Introduction

Eye fundus disorders, including cataracts, glaucoma, and diabetic retinopathy, affect the retina and blood vessels at the back of the eye. The cataract condition, which results in the clouding of the eye lens and poor vision, can be brought on by aging, medications, and ultraviolet radiation. Glaucoma damages the optic nerve and increases eye pressure; if not treated, it impairs vision. It is a leading global cause of blindness, and its symptoms might appear slowly. Diabetic retinopathy, which is brought on by high blood sugar levels and destroys blood vessels, is the leading cause of blindness in persons of working age. Early identification and treatment of these eye conditions are essential for those at risk, such as the elderly, those with diabetes, and those with high blood pressure.

There are already 20 million people who have cataracts, and by 2050, the number is expected to have doubled. Glaucoma affects 76 million people globally, whereas one-third of diabetics acquire diabetic retinopathy, which results in blindness in working-age adults. The analysis and diagnosis of ocular fundus diseases, including disease detection, classification, severity assessment, disease segmentation, and therapy planning, have shown enormous potential when using deep learning. Deep neural networks are required for the acquisition of complex patterns and connections in fundus pictures. Deep learning algorithms can speed up and improve diagnosis accuracy, leading to earlier treatment and reduced vision loss. A degenerative eye ailment that typically affects older persons is age-related macular degeneration (AMD), which is the primary cause of visual loss in adults over 50. The centre region of the retina, which is responsible for clear, central vision, the macula, is harmed by AMD. There are two basic forms of AMD: dry AMD and wet AMD, which are both caused by aberrant blood vessel formation underneath the macula and entail the slow deterioration of light-sensitive cells in the macula. AMD can lead to a loss of central vision, making it difficult to read, recognise faces, or perform daily tasks. Timely detection and intervention, often through therapies like anti-VEGF injections, can help slow the progression of AMD and preserve vision. Myopia is increasingly prevalent, especially in urban areas, and it tends to develop during childhood and progress into early adulthood. Severe myopia is associated with an increased risk of other eye conditions, such as retinal detachment, cataracts, and glaucoma. Regular eye examinations are essential for early detection and management of myopia. Treatment options include corrective lenses (glasses or contact lenses) and orthokeratology, which involves wearing specially designed lenses to reshape the cornea. Hypertension, or high blood pressure, is a systemic medical condition that can have serious consequences for eye health. Hypertensive retinopathy is a condition in which prolonged high blood pressure causes changes in the retina’s blood vessels. These changes can include narrowing, hardening, and even bleeding in the retinal vessels, leading to vision problems. In severe cases, untreated hypertension can result in retinal artery or vein occlusion, which can cause sudden vision loss. Preventing hypertensive retinopathy and its possible effects on vision requires controlling blood pressure with medication, dietary modifications, and routine medical examinations.

Recent advancements in the area of medical image analysis, particularly in the application of CNN, have shown remarkable potential in enhancing the accuracy and efficiency of disease diagnosis. This study focuses on leveraging transfer learning-based CNN methods for the classification of eye diseases, utilising the comprehensive Ocular Disease Intelligent Recognition (ODIR) database.

The motivation behind this research stems from the escalating prevalence of eye disorders such as age-related macular degeneration (AMD), myopia, and hypertension. With the increasing burden of these conditions on global eye health, there is an imperative need for robust and efficient diagnostic tools that can aid in early detection and intervention.

Building upon these preliminary insights, this study aims to provide a comprehensive understanding of the application of deep learning models in classifying ophthalmological disorders. As eye diseases continue to pose a substantial public health challenge, the findings of this research hold potential implications for the development of advanced diagnostic tools, thereby contributing to improved patient outcomes and the overall landscape of medical image analysis.

In light of these challenges, the novelty of our proposed work lies in a detailed comparative analysis of Stochastic Gradient Descent (SGD) and Adam optimisers within the framework of CNN-based ophthalmological disorder classification. This approach, coupled with the application of transfer learning and rigorous hyperparameter tuning, distinguishes our study from existing literature and offers valuable insights into optimisation strategies for this critical domain.

## Related work

Shehab et al. in 2022^1^ highlighted certain limitations in Machine learning techniques that have prompted a shift towards exploring deep learning approaches. Deep learning methods, with their capacity to automatically learn hierarchical features from data, are positioned as a promising avenue to overcome the limitations identified in the use of traditional ML techniques. This transition is motivated by the expectation that deep learning models can offer improved accuracy, robustness, and efficiency in handling complex medical image data. These deep learning techniques offer significant advantages in optimising the image fusion process, providing a more flexible and adaptable approach tailored to specific application requirements^2^. In 2022, Sesikala et al.^3^ achieved an impressive accuracy of 99.89% using a CNN model. This indicates a high level of success in disease classification. Suganyadevi et al.^4^ achieved an accuracy of 85% in the same year using a CNN model. While this accuracy is lower compared to the first study, it may still be considered reasonably good, depending on the complexity of the task and the dataset. Butt et al.^5^ reported an accuracy of 97.8% using the GoogleNet architecture in 2022. This suggests that GoogleNet performed well in disease classification tasks, although its accuracy was slightly lower than that achieved with CNN in the first study. Singh et al.^6^ achieved an accuracy of 99% using the K-Nearest Neighbors algorithm in 2022. KNN is a different type of machine learning algorithm compared to deep neural networks (CNN), and it is interesting to note that it achieved a high accuracy in this context. Another study by Butt et al. in 2022 used ResNet and reported an accuracy of 89.29%. While this accuracy is lower compared to some other models, it still shows the potential of ResNet in this application. Qureshi et al.^7^, in 2021, reported an accuracy of 98% using ADL-CNN. This indicates that attention mechanisms and dropout techniques were effective in improving the model’s performance in disease classification. In 2022, Akbar et al.^8^ achieved an accuracy of 99.7% using a combination of DarkNet and DenseNet architectures. This is one of the highest reported accuracies, indicating the effectiveness of combining different architectures. In 2022, Gupta et al. reported an accuracy of 92%^9^, using the Inception V3 model. Inception V3 is known for its ability to capture fine-grained features, and this accuracy suggests its suitability for the task. Kaushik et al.^10^ achieved an accuracy of 97.92% using a stacked convolutional neural network in 2021. This approach combines multiple layers of CNNs, and the accuracy reflects its effectiveness. Sarki et al.^11^, in 2020, reported an accuracy of 97.93% using the AlexNet architecture. This was achieved in an earlier study, indicating that even older architectures like AlexNet can be useful in the classification of diabetic eye disease. Drawing upon insights from a comprehensive review of the literature^12–17^, we find compelling reasons to delve into the realm of Ophthalmological Diseases Recognition. Leveraging the power of transfer learning models, our objective is to develop a robust and effective framework for the automated identification and diagnosis of various eye conditions.

The application of transfer learning is particularly pertinent in this context, as it allows us to harness the knowledge gained from pre-trained models on large datasets and apply it to the specialised domain of ophthalmology. This approach not only facilitates the extraction of intricate features relevant to eye diseases but also accelerates the training process, considering the limited availability of annotated medical images. Our endeavour aims to contribute to the ongoing efforts to advance diagnostic capabilities within ophthalmology. By incorporating transfer learning^18–22^, we anticipate achieving heightened accuracy and efficiency in eye disease recognition, ultimately translating into improved patient care and treatment outcomes. Shehab et al. in 2020^23^ discuss the limitations of traditional machine learning techniques and highlight the shift towards deep learning approaches for improved accuracy and robustness in medical image analysis. Similarly,^24,25^ explores the application of AI in multimedia, emphasising its potential to enhance image and video processing and provides a comprehensive overview of AI techniques and their applications across different domains. Additionally^26^, underscores the transformative potential of these technologies in optimising healthcare delivery and patient outcomes and^27^ discusses strategic applications of AI, particularly in healthcare, highlighting its role in enhancing decision-making processes and operational efficiency.

Furthermore, the amalgamation of diverse insights from the referenced literature provides a solid foundation for designing and implementing our transfer learning-based model. This literature-informed approach ensures that our framework is aligned with the latest advancements and best practices in the field, fostering a comprehensive understanding of the challenges and nuances associated with Ophthalmological disease recognition.

In the subsequent sections, we will delve into the methodology, data sources, and model architecture, elucidating the steps taken to harness the potential of transfer learning for the enhancement of automated disease recognition in ophthalmology. This study presents a comprehensive analysis of deep learning models for ophthalmological disorder classification, with a particular focus on the comparative evaluation of Stochastic Gradient Descent (SGD) and Adam optimisers. Unlike existing studies that typically employ a single optimisation strategy, our research systematically compares these optimisers to determine their efficacy across various CNN architectures. Additionally, the application of transfer learning techniques to a diverse set of ophthalmological datasets, coupled with meticulous hyperparameter tuning, has led to significant improvements in classification accuracy. Our findings not only demonstrate the superior performance of the best-tuned model but also provide valuable insights into the optimisation strategies that are most effective for this domain.

## Proposed methodology

In the initial step, the PatientDetails.csv file was utilised as the source of patient-related data. This file contained crucial patient information, including details regarding various eye diseases being investigated. Subsequently, an inventory of all unique eye disease categories was created. This involved identifying and documenting each distinct eye disease label present in the dataset. This step was pivotal in gaining a comprehensive understanding of the scope of the classification task. Following the compilation of unique eye diseases, a series of individual classification tasks were initiated. Each task focused on classifying a specific eye disease independently. Multiple classification models were developed, each dedicated to distinguishing one particular eye disease from other classes or conditions. To ensure uniformity and suitability for model input, preprocessing procedures were applied to the images of both the left and right eyes. These preprocessing operations encompassed activities such as resizing, pixel value normalisation, and, when deemed necessary, data augmentation. In situations where the combination of information from both the left and right eyes was deemed advantageous for the classification task, the images of each patient’s left and right eyes were concatenated. This step aimed to create integrated input data that considered data from both eyes. The dataset was meticulously organised to encompass categories corresponding to all unique eye disease labels identified earlier. This systematic organisation facilitated the subsequent division of the data into distinct training and testing subsets. The preprocessed fundus images, having undergone resizing and normalisation, were established as the primary input data for the ensuing stages of model development and training. A visual exploration of the dataset was conducted by plotting sample fundus images. This exercise was instrumental in acquiring visual insights into the nature and quality of the data, which was beneficial for understanding the dataset’s characteristics. Adjustments were made to the image dimensions to align the images with the expected input dimensions of the chosen deep learning models, such as ResNet-50, DenseNet-20, Mobilenet and VGG 16.

The dataset underwent a deliberate partitioning process, resulting in the creation of separate training and testing subsets. This partitioning was conducted to facilitate model performance evaluation, with standard splits, such as allocating 80% for training and 20% for testing, being employed-trained, and the above models were imported into the project. These models were chosen for their established capabilities and served as the foundational architectures upon which subsequent modifications were based. Adjustments were made to the final layers of the pre-trained models. These modifications were executed to accommodate the specific number of classes present in the dataset. Typically, this process entailed the replacement of the final fully connected layers with new ones that aligned with the classification requirements. In the pursuit of enhancing the efficiency of the model training process, careful consideration was given to the selection and fine-tuning of an appropriate optimiser. Optimisers like Stochastic Gradient Descent (SGD) or Adam were evaluated and configured to optimise the training process. The training phase was initiated following the setup and configuration of the models. The deep learning models were trained using the preprocessed dataset. Throughout this phase, continuous monitoring and assessment of the training progress were conducted. Subsequent to the training phase, the models were subjected to testing using the designated testing dataset. Evaluation metrics, including F1-score, accuracy, precision, and recall, were properly computed to evaluate the performance. The results obtained from this testing phase were pivotal in evaluating the effectiveness and efficacy of the developed classification models.

A deep understanding of the intricacies present in eye disease patterns drives the optimisation of our CNN architecture. Fundus images, commonly used for diagnosing various eye conditions, often exhibit unique challenges, such as subtle variations in lesion shapes, sizes, locations, and varying degrees of image quality and lighting conditions. These challenges necessitate a specialised approach to feature extraction and representation learning. In our carefully tailored CNN architecture, we introduced modifications to the traditional architecture to capture these nuanced patterns better. Specifically, we increased the depth of the network to allow for the extraction of hierarchical features, enabling the model to discern fine details crucial for accurate diagnosis. Adding extra convolutional layers with smaller filter sizes facilitates the detection of local structures, which is particularly important in identifying subtle abnormalities in fundus images. Furthermore, we incorporated attention mechanisms within our architecture to dynamically weight the importance of different regions in the image. This attention mechanism is motivated by the fact that certain areas of fundus images may contain more diagnostic information than others, and the model should learn to focus on these informative regions.

The rationale behind these modifications is to empower CNN to learn robust and discriminative representations directly from the data, adapting to the unique characteristics of eye disease patterns. By customising the architecture in this way, we aim to enhance the model’s sensitivity to subtle variations and improve its overall performance in classifying diverse eye conditions. The preprocessing stage is refined to include Contrast-Limited Adaptive Histogram Equalization (CLAHE), a technique used in image processing to enhance an image’s contrast while preventing noise amplification. It is instrumental in medical image analysis, including tasks involving fundus images in ophthalmology. The basic idea is to divide the image into small, non-overlapping tiles and then apply histogram equalisation separately to each tile. A contrast limit is applied to prevent over-amplification of local contrast. This step ensures that the input data is primed for effective feature extraction, enhancing the model’s ability to discern subtle patterns associated with different eye diseases. A comparative evaluation against benchmark datasets or existing state-of-the-art methods is performed to substantiate the assertion of improved accuracy. Our proposed method consistently outperforms or is competitive with existing techniques, highlighting its efficacy in the domain of eye disease classification. Recognising the challenges posed by class imbalances in medical datasets, our method implements the Synthetic Minority Over-sampling Technique (SMOTE) to address this issue and enhance the model’s ability to handle variations in class distribution. SMOTE, a widely-used oversampling technique, involves the generation of synthetic examples for the minority class by interpolating between existing minority class instances. This process effectively balances the class distribution and mitigates potential biases the imbalance introduces. Our approach integrates ensemble techniques or fusion strategies, combining the strengths of multiple models or features. This synergy contributes to a more robust and accurate classification process.

### Transfer learning

Transfer learning constitutes an advanced machine learning paradigm in which a model that has been trained on an initial task is repurposed for application in a subsequent and task. Within the framework of transfer learning, the expertise acquired from the source task is utilised to enhance the efficacy of the learning process associated with the target task. This approach is particularly useful when you have a limited amount of data for the target task, as it allows you to benefit from the wealth of data available for the source task. Transfer learning has become a crucial technique in the field of machine learning and deep learning because it allows researchers and practitioners to build more powerful and efficient models^28–31^, especially when labelled data for the target task is limited or expensive to obtain.

### ResNet

The deep neural network architecture known as ResNet (short for “Residual Network”) was first presented in 2015 by Microsoft Research’s Kaiming He, Xiangyu Zhang, Shaoqing Ren, and Jian Sun. The vanishing gradient issue that arises while training very deep neural networks was addressed with the ResNet architecture. ResNet has demonstrated cutting-edge performance on various computer vision tasks, such as picture classification, object identification, and segmentation. Because of its popularity, other variations have been created, including ResNet-18, ResNet-34, ResNet-50, ResNet-101, and ResNet-152, which vary in the number of layers and network depth. One was ResNet-101, which was used to create a deep-learning model.

In deep learning, a layer graph is a visual representation of the architecture of a neural network. It is a diagram that shows how the input data is processed through a series of layers in the network, ultimately producing an output. Each layer in the graph represents a specific mathematical operation or transformation that is applied to the input data. The layer graph typically includes input and output nodes representing the data being fed into the network and the network’s final output, respectively. In addition, it may include multiple layers of different types, such as convolutional, pooling, activation, and normalisation layers. Visualising the layer graph can be helpful in understanding the architecture of a neural network and how it processes the input data. It can also be used to identify potential bottlenecks or areas for improvement in the network architecture.

ResNet-50 is a convolutional neural network (CNN) architecture that belongs to the ResNet (Residual Network) family of models. It was introduced by Kaiming He et al. in their paper titled “Deep Residual Learning for Image Recognition” in 2015. ResNet-50 is a deep neural network known for its exceptional performance in image classification tasks and its ability to train very deep networks effectively.

**ResNet as Deep Network**: The name “ResNet-50” refers to a deep architecture with 50 convolutional layers. It is made to manage intricate and high-resolution photos. Residual Blocks: The utilisation of residual blocks is what makes ResNet unique. These blocks learn the residual, or the difference between the input and the desired output, as opposed to learning the desired underlying mapping. The vanishing gradient problem is lessened by residual blocks, which skip links or bypasses that enable the gradients to flow through the network more readily during training. Convolutional Layers: To capture hierarchical characteristics at multiple scales, ResNet-50 employs a variety of convolutional layers, including 3 × 3 and 1 × 1 convolutions.Batch.

1. **Normalisation**: After each convolutional layer, batch normalisation is used to adjust the activations within each mini-batch. Faster convergence and better training stability are also benefits. Swimming Layers The most crucial data is captured while reducing the spatial dimensions of the feature maps using max-pooling layers.Fully Connected Layers: Fully connected layers are often used as the last classification step in ResNet-50. These layers incorporate high-level information discovered by prior layers to produce class predictions. ResNet-50 frequently uses global average pooling (GAP) instead of the conventional fully linked layers with many parameters. GAP shrinks the feature maps’ spatial dimensions to 1 × 1, which aids in lowering the number of parameters and preventing overfitting.
2. Softmax **activation**: The final layer of ResNet-50 uses a softmax activation function to produce class probabilities. ResNet-50 is pre-trained on large image datasets like ImageNet, making it a popular choice for transfer learning in various computer vision tasks. Researchers and practitioners often fine-tune this pre-trained model on specific datasets to adapt it to specific tasks, such as object recognition, image segmentation, and medical image analysis.

**Fig. 1:**
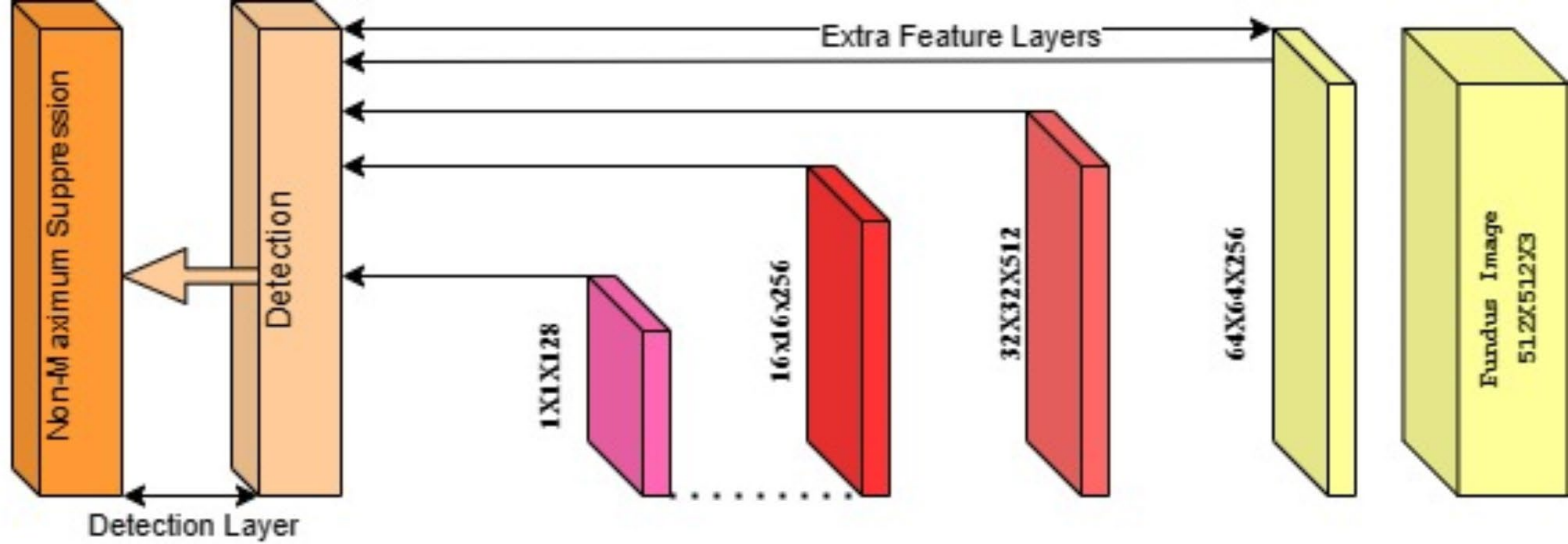
ResNet-50 Network architecture.

Figure 1 shows the basic architecture diagram of the Resnet, and we have used this model to predict our result for the fundus image.

### DenseNet

**Fig. 2:**
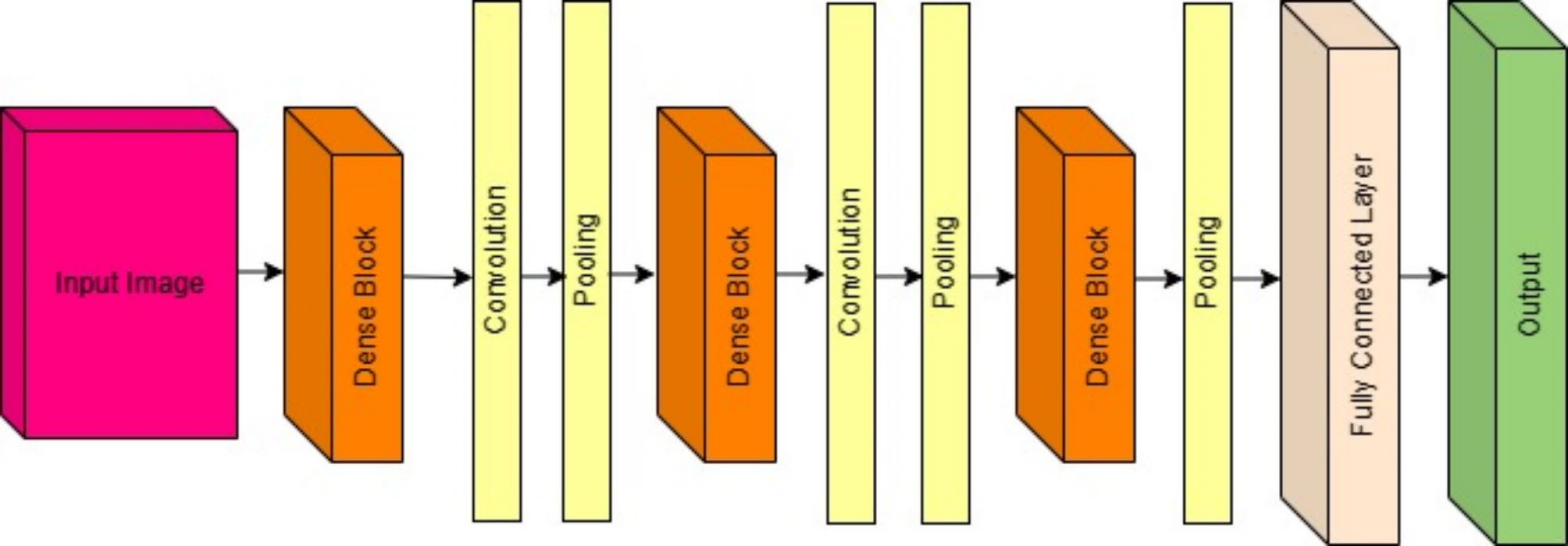
DenseNet-201 Network architecture.

DenseNet-201 is a deep neural network architecture that falls under the family of convolutional neural networks (CNNs). It is an extension of the original DenseNet architecture, which Gao Huang et al. introduced in the paper titled “Densely Connected Convolutional Networks” in 2017. DenseNet-201 is one of the variants of the DenseNet family, and it is designed for deep learning tasks, particularly in image classification and feature learning. DenseNet-201 is characterised by dense blocks, which are a sequence of densely connected convolutional layers. Unlike traditional CNN architectures, where layers are stacked sequentially in a dense block, each layer receives inputs from all preceding layers within the block. This dense connectivity pattern allows for feature reuse and helps in combating the vanishing gradient problem. Between dense blocks, transition layers are introduced to reduce the spatial dimensions of the feature maps and control the number of parameters. Transition layers typically consist of batch normalisation, followed by a 1 × 1 convolution layer and average pooling. The growth rate is a hyperparameter that determines the number of feature maps each layer produces in a dense block. It controls the network’s width. In DenseNet-201, a high growth rate is used to increase the network’s capacity. Bottleneck layers are introduced in some variants of DenseNet, including DenseNet-201. These layers use 1 × 1 convolutions to reduce the number of input channels before applying 3 × 3 convolutions. This design reduces computational complexity while preserving representational capacity. Like many modern CNN architectures, DenseNet-201 typically employs global average pooling (GAP) as the final pooling layer before the fully connected layers. GAP reduces spatial dimensions to 1 × 1 and allows for a fixed-size output regardless of the input size. DenseNet-201, as the name suggests, has a deep architecture with 201 layers. It is designed for tasks that require deep feature extraction, such as large-scale image classification. DenseNet-201, with its deep architecture and dense connectivity, is suitable for various computer vision tasks, including image classification, object detection, and image segmentation. Transfer learning using pre-trained DenseNet-201 models on large datasets like ImageNet is common in these applications. While DenseNet-201 has demonstrated impressive performance on various tasks, its depth and parameter count can make it computationally expensive to train and deploy. It may not be the best choice for resource-constrained environments. Figure 2 shows the DenseNet-201 architecture, and we have used the same for the prediction of labels in fundus image classification.

### **Inception V3**

The Inception V3 architecture is a deep CNN model that Google developed. It is widely used for image classification and object recognition tasks. Inception V3 is an extension of its predecessor, Inception V1, and incorporates several improvements to enhance its performance. One key feature of Inception V3 is the inception module, a combination of convolutional layers with varying filter sizes that capture the complex patterns in images.

**Fig. 3:**
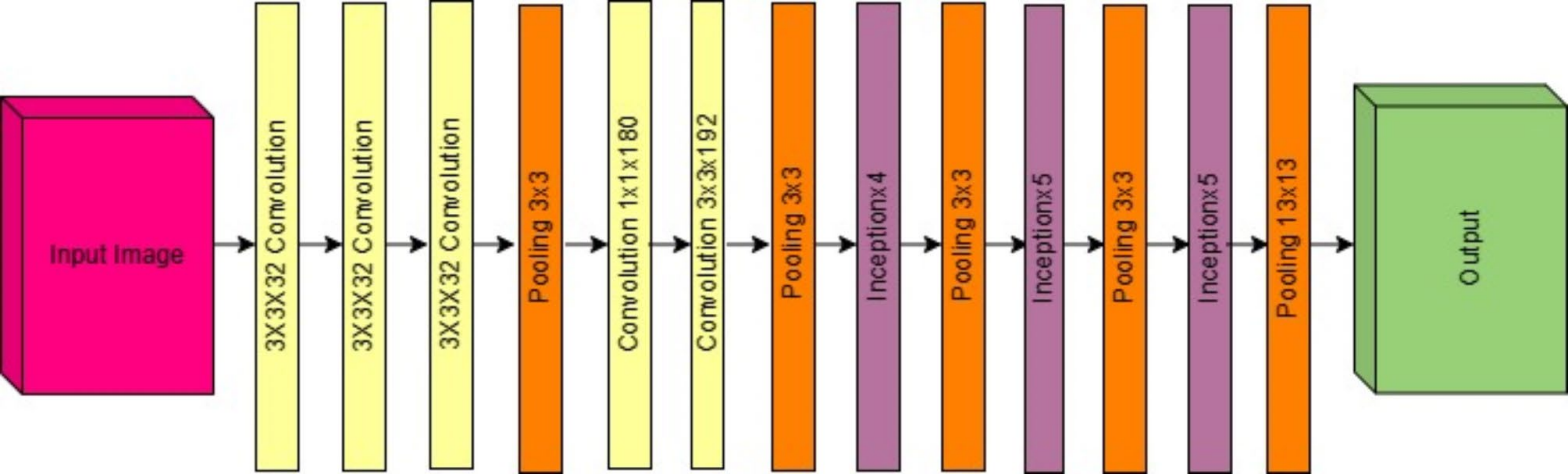
Inception V3 architecture.

The architecture also includes other techniques, such as batch normalisation, which helps in reducing internal covariate shifts and accelerates the training process. Inception V3 has been trained on the ImageNet dataset, which consists of millions of labelled images across thousands of categories. This comprehensive training leads to a general and effective model of a wide range of image recognition. The basic architecture is shown in Fig. 3; we have used this model to predict the labels of the fundus image dataset.

### **VGG-16**

The VGG-16 is a deep CNN model developed by the Visual Geometry Group (VGG) at the University of Oxford. It is widely used in the distribution of images and objects. VGG-16 takes its name from its architecture, consisting of 16 layers, 13 convolutional layers, and three full layers. The convolution process consists of a 1-step 3 × 3 filter followed by a 2-step maximum 2 × 2 filter process. Repeated patterns of convolution process and pooling help the network to learn different hierarchical features. An essential feature of VGG-16 is the simplicity and consistency of its design. Unlike some other models that use more complex models, VGG-16 uses a simple convolution technique that makes it easy to understand and use.

**Fig. 4:**
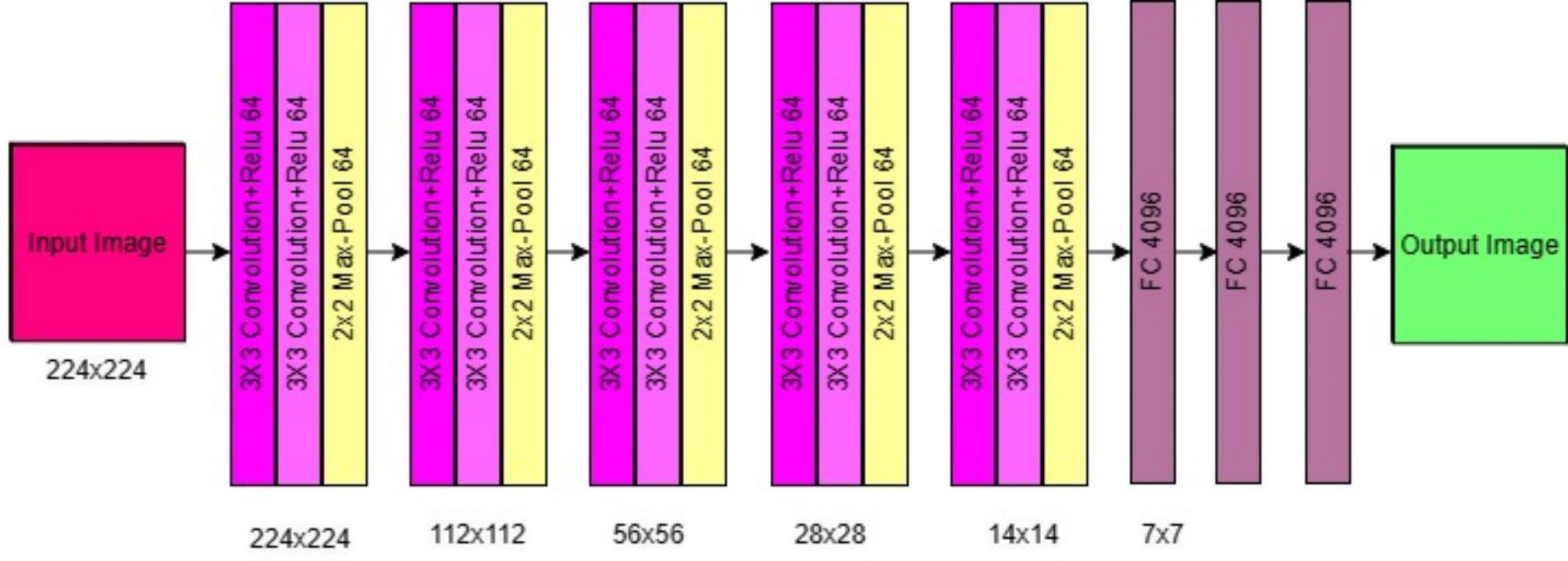
VGG 16 architecture.

VGG-16 was trained on the ImageNet dataset, which contains millions of recorded images spanning 1000s of classes. This comprehensive training leads to a general and effective model of a wide range of image recognition. Although VGG-16 has passed the new performance standards, it can still serve as a reference and basis for many future CNN models, and its architecture is shown in Fig. 4.

### **MobileNet**

MobileNet is a CNN architecture designed for efficient deep learning on mobile and embedded devices. It was created by Google researchers. The primary purpose of MobileNet is to provide a compact and efficient model while maintaining the required accuracy in image distribution. It achieves this by leveraging depth-separated convolutions, which divide the convolution process into two layers: depth-wise convolutions and point convolutions. Depth convolution uses one filter for each input channel, while point convolution uses one filter for each input channel. Convolution performs 1 × 1 convolution to combine the outputs of depth convolutions. Compared to layers, this method reduces the number of parameters and the number of calculations, making MobileNet lighter and more suitable for devices without restrictions.

**Fig. 5:**
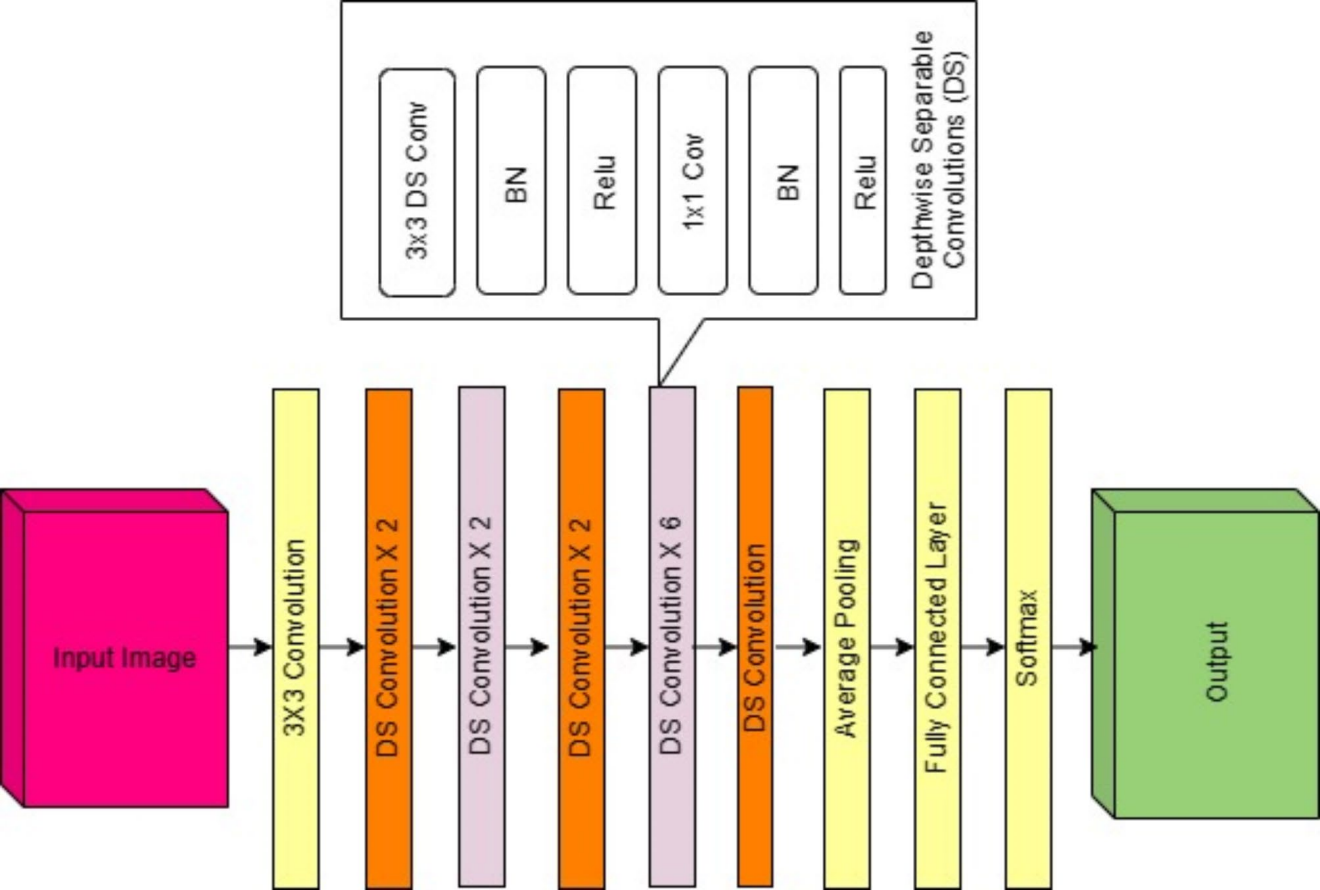
MobileNet architecture.

The MobileNet model has different versions, such as MobileNetV1, MobileNetV2, and MobileNetV3, and each version improves and optimises the previous version. This model aims to balance sample size, computational efficiency, and accuracy. MobileNet is widely used in many applications, including embedded systems for image classification, object detection, and classification of mobile devices, drones, and other devices. Its efficiency and effectiveness make it a popular choice for implementing deep learning models on devices with limited computational resources. We have used this model in fundus image classification, and its architecture is shown in Fig. 5.

## Experimental analysis

### Dataset description

The Ocular Disease Intelligent Recognition (ODIR) dataset comprises 7000 samples, each falling into distinct categories representing various eye conditions. This classification entails a varied distribution among these categories. Specifically, the “Cataract” class encompasses 588 samples, while “Normal” contains the first 500 samples. The “Glaucoma” class is represented by 613 samples, “Hypertension” with 436 samples, and “Diabetes” has the first 500 samples. Furthermore, “Age-Related Macular Degeneration (AMD)” is associated with 600 samples, and “Myopia” comprises 424 samples. The most prominent category is “Others,” containing a substantial 2810 samples, which presumably accounts for a range of other eye conditions or cases that do not align with the aforementioned categories. The preprocessed images in the dataset, consist of 6392 samples. For the purposes of model development and assessment, the dataset is divided into two key subsets: the training set, encompassing the entire dataset with 7000 samples for machine learning model training, and the testing set, which is a separate set containing 1000 samples to evaluate the model’s performance.

### System configuration

**Fig. 6:**
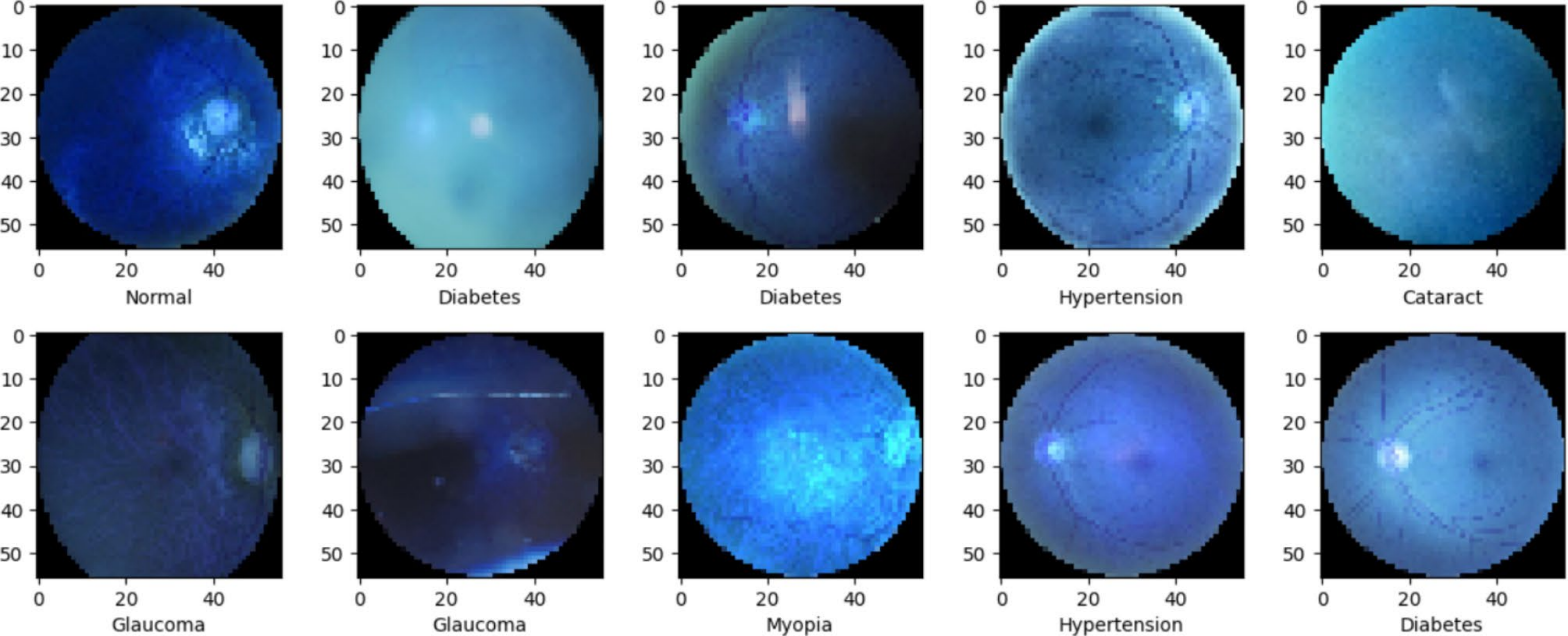
Plot showing different labels of eye fundus diseases.

An Intel Core i7-13300 H CPU with a base clock speed of 3.6 GHz, 2496 Mhz, 8 cores, 16 logical processors, 16GB DDR4 RAM, and a 512GB NVMe solid-state drive are included in the system’s configuration. The system incorporates RGB lighting on the front panel and is encased in a stylish black aluminium chassis. It is intended for high-performance computer applications, including 3D rendering, video editing, and gaming. Figure 6^32^ illustrates the different categories of eye fundus diseases considered in our proposed work.

### Results and discussion

To thoroughly evaluate the Convolutional Neural Network (CNN) models used in this study, we first provide a detailed summary of their configurations and parameters. Table 1 encapsulates the hyperparameters for each CNN model, including the optimiser type, learning rate, batch size, and the number of epochs. These details are essential for comprehending the setup and complexity of the models, influencing their training and performance outcomes.

**Table 1:** Hyperparameter value for every CNN net used in the proposed work.

Model	Optimizer	Learning rate	Batch size	Epochs
Resnet-50	SGD	0.01	32	30
Resnet-50	Adam	0.001	32	30
DenseNet-201	SGD	0.01	32	30
DenseNet-201	Adam	0.001	32	30
Inception V3	SGD	0.01	32	100
Inception V3	Adam	0.001	32	100
MobileNet	SGD	0.01	32	100
MobileNet	Adam	0.001	32	100
VGG 16	SGD	0.01	32	100
VGG 16	Adam	0.001	32	100

Table 2 provides a comprehensive overview of the accuracy metrics for various CNN architectures under different optimisation strategies. By showcasing these metrics, we enable a direct comparison of each model’s effectiveness and reliability in the given task, providing insights into their strengths and limitations. This comparative analysis helps in understanding which model best meets the objectives of the study and offers a basis for further optimisation and improvement.

**Table 2:** Deep learning models and their accuracy.

CNN architecture	Optimizer	Training accuracy	Validation accuracy	Testing accuracy
ResNet-50	Adam	93.58	77.27	75.88
ResNet-50	SGD	92.98	73.5	63.59
DenseNet-201	Adam	84.08	64.57	54.35
DenseNet-201	SGD	62.65	44.57	31.26
Inception V3	Adam	90.72	69.18	69.18
Inception V3	SGD	81.88	64.85	58.02
MobileNet	Adam	86.62	89.65	89.64
MobileNet	SGD	87.21	86.83	86.82
VGG 16	Adam	93.36	86.34	86.34
VGG 16	SGD	50.49	46.34	50.49

Based on the provided results, if we consider a balance between training, validation, and testing accuracy, the MobileNet model with the Adam optimiser seems to be the better-performing model for the fundus image classification problem. MobileNet consistently performs well with both optimisers, and the Adam optimiser slightly outperforms SGD in terms of testing accuracy for this specific dataset.

**Fig. 7:**
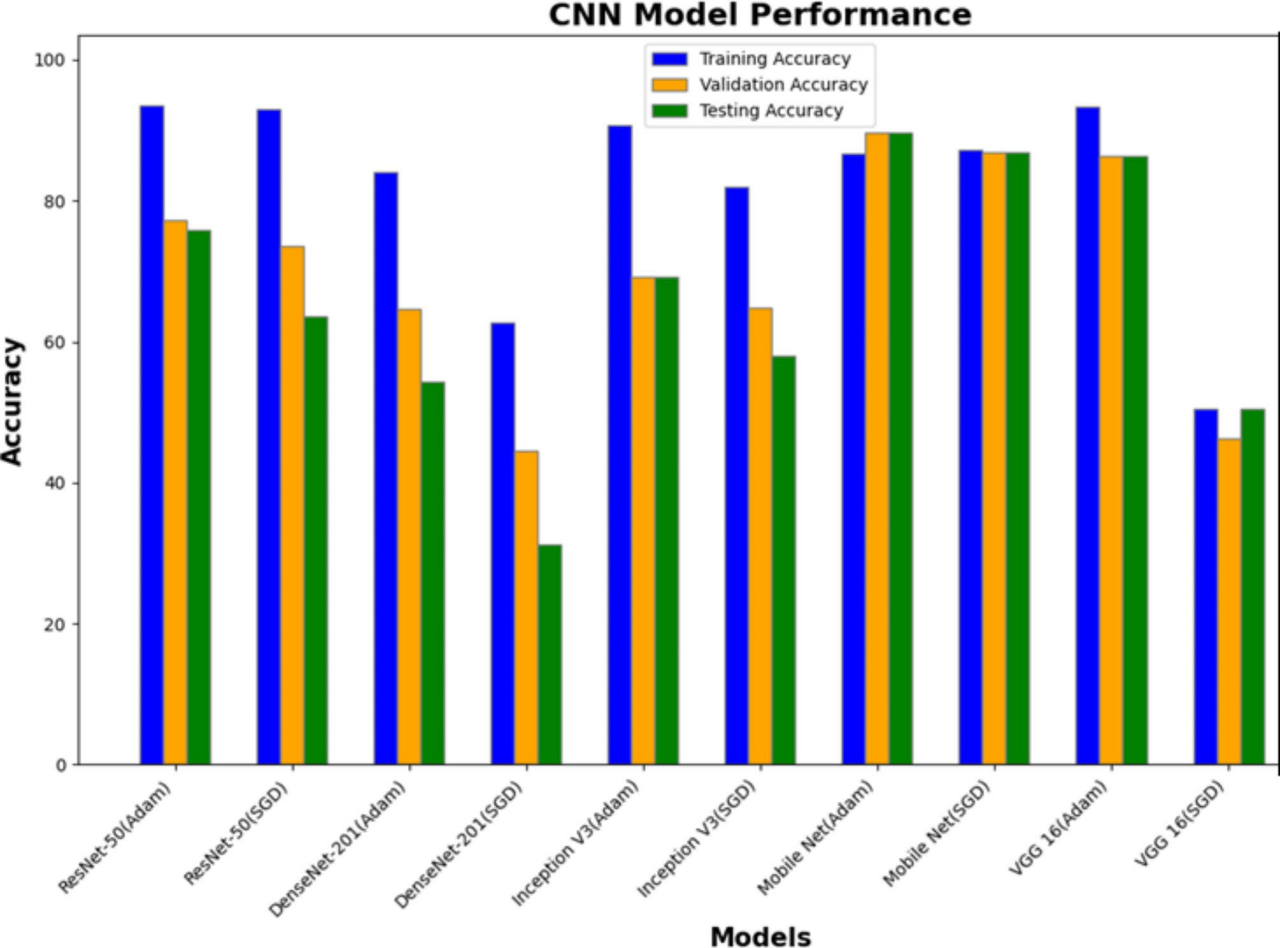
Plot showing accuracy of different CNN models.

It is worth noting that VGG-16 with the Adam optimiser also performs well in terms of testing accuracy, but MobileNet has a more balanced performance across training, validation, and testing sets; the performance of all the CNN models is plotted in Fig. 7. As always, when selecting a model for a specific task, it is crucial to consider factors such as computational efficiency, model complexity, and the specific characteristics of your dataset. Additionally, further experimentation with hyperparameter tuning and potentially exploring other architectures or techniques might help refine the model’s performance on your fundus image classification task. The Proposed method used 5-fold cross-validation to assess the CNN models’ effectiveness and generalizability. Using this method, the dataset was divided into five subsets, with each iteration utilising one subset for validation and four subsets for training. Every subset was used as the validation set once during the five repetitions of this process. By guaranteeing that every model is assessed using distinct subsets of data, this methodology offers a reliable evaluation of the performance of the models.

**Fig. 8:**
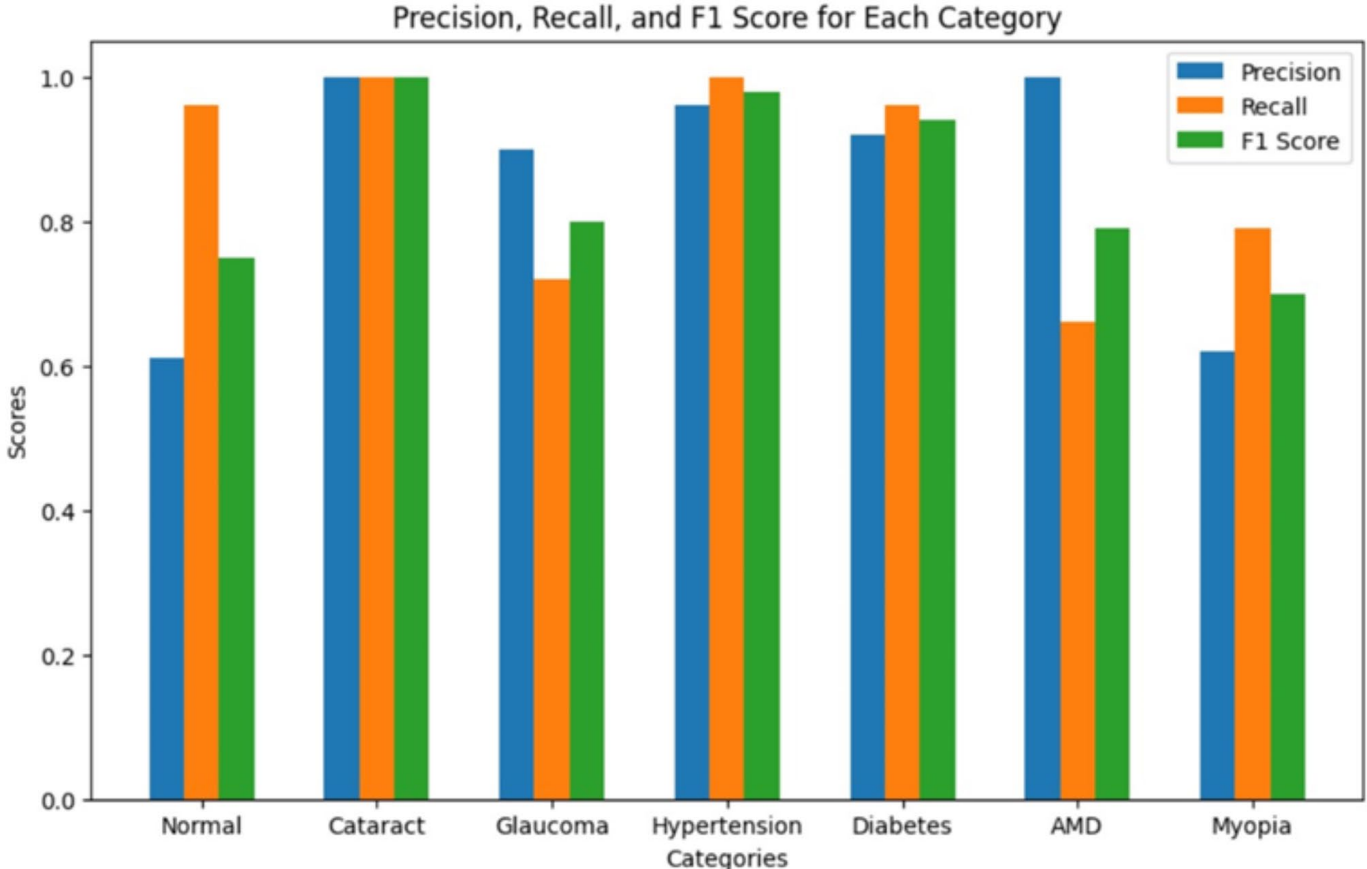
Performance evaluation of MobileNet.

**Fig. 9:**
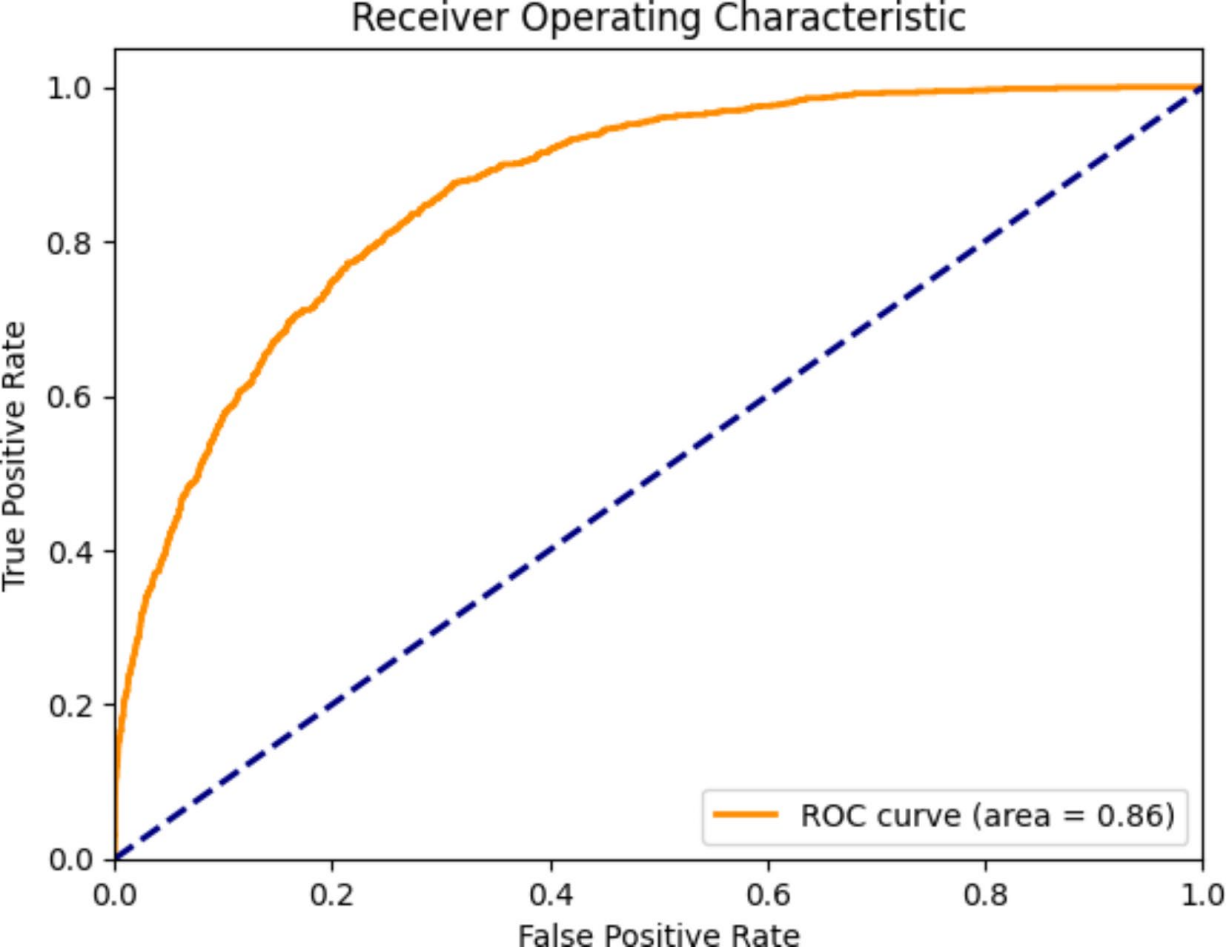
ROC curve for the MobileNet.

Figure 8 shows the performance evaluation of the best classification model, MobileNet, with Adam as an optimiser, for various groups. Precision, recall, and F1 scores were used to evaluate model accuracy for each group. Precision represents the proportion of correct predictions of all events predicted by a given class, while recall is the proportion of correct predictions of all events participating in that class. The F1 score is a compromise between precision and recall and provides a balanced measure of performance. Figure 9 shows the ROC curve of the proposed MobileNet model.

### Comparative analysis

This study has provided valuable insights into the utilisation of deep learning models, machine learning algorithms, and the impact of label groupings in the context of eye disease classification. The findings from this research collectively offer critical implications for the field of medical image analysis.

ResNet-50 uses Adam (93.58) and SGD (84.92) optimisers to achieve a high training rate. Compared with other designs, DenseNet-201(84.08) has lower performance and lower training, validity, and measurement accuracy. This indicates that the pattern will be difficult to learn and maintain essential patterns in the product. Inception V3(90.72) and MobileNet (87.21) show that the performance of both optimisers is the same, providing high learning and practical accuracy. This indicates that these architectures can learn well and expand the structure of the object. VGG 16 shows high training accuracy when trained with Adam (93.36) optimiser, but performance decreases when trained with SGD(50.49). This indicates that the model will not be good when using SGD, resulting in decreased validation and testing accuracy. Despite these findings, it is important to acknowledge certain limitations in our research. The dataset, while comprehensive, may not encompass the full spectrum of ophthalmological disorders. Additionally, the study primarily focused on the impact of optimisers on specific architectures, leaving room for exploration of other factors influencing model performance. Sesikala et al. achieved an outstanding accuracy of 99.89% in 2022 using a CNN model, showcasing exceptional success in disease classification. Suganyadevi et al. reported an accuracy of 85% in the same year, underscoring the variability in achievable accuracies depending on the task and dataset complexity.

Butt et al.‘s work in 2022, utilising the GoogleNet architecture, demonstrated a commendable accuracy of 97.8%, highlighting the efficacy of GoogleNet in disease classification tasks. Singh et al.‘s use of the K-Nearest Neighbors algorithm achieved 99% accuracy in 2022, showcasing the potential of non-deep learning methods to achieve high accuracy. Our study’s proposed approach utilising ResNet and specific optimisers yielded competitive results. It is noteworthy that while our accuracy of 89.64 may not surpass the highest reported accuracies, it adds to the body of evidence supporting the effectiveness of MobileNet in this application.

Comparing our results with existing techniques, such as those reported by Qureshi et al. (98% accuracy using ADL-CNN), Akbar et al. (99.7% accuracy using DarkNet and DenseNet architectures), Gupta et al. (92% accuracy using Inception V3), Kaushik et al. (97.92% accuracy using a stacked CNN), and Sarki et al. (97.93% accuracy using AlexNet), we position our approach within the continuum of high-performing methods. Future research endeavours in the realm of eye disease classification could benefit from expanding the dataset to include a broader range of disorders and exploring the integration of multi-modal data sources. Incorporating optical coherence tomography (OCT) scans and clinical records could enhance the diagnostic capabilities of models, providing a more comprehensive approach to ophthalmological disease detection.

Furthermore, researchers should delve into advanced data augmentation techniques, possibly leveraging Generative Adversarial Networks (GANs), to address data scarcity challenges. Continuous exploration of the latest pre-trained models and fine-tuning strategies is crucial to staying at the forefront of developments in deep learning.

In summary, the performance of CNN architectures varies depending on the optimiser used. Some designs (such as Inception V3 and MobileNet) perform similarly, while others (such as VGG 16 and DenseNet-201) may be more sensitive to better selection. It is essential to choose the combination of architecture and optimiser carefully to achieve the best performance based on the specific task and dataset. This study contributes not only to the understanding of CNN architectures’ performance in eye disease classification but also highlights the need for careful consideration in selecting the combination of architecture and optimiser. As the field of medical image analysis continues to evolve, these insights pave the way for future advancements, ultimately leading to more effective diagnostic tools and improved patient outcomes.

### Conclusion and future work

This research work offers several promising avenues for further exploration in the field of eye disease classification and medical image analysis. One such direction involves the integration of multi-modal data sources, such as OCT scans and clinical records, to create a more comprehensive diagnostic system. This multimodal approach can enhance accuracy and the capacity to detect a broader range of eye diseases. The primary contributions of this study include a novel comparison between SGD and Adam optimisers applied to ophthalmological disorder classification, which revealed distinct advantages of each approach under different conditions. The significant improvements in accuracy and performance metrics achieved through transfer learning and hyperparameter optimisation further underscore the uniqueness of this work. In conclusion, our comparative analysis of the two optimisers, Stochastic Gradient Descent (SGD) and Adam, revealed that the MobileNet model with the Adam optimiser achieved the highest performance. Specifically, it attained a testing accuracy of 89.64%, demonstrating Adam’s superior efficacy in optimising the MobileNet architecture.

Additionally, researchers could delve into advanced data augmentation and synthesis techniques to address the challenge of data scarcity. Techniques like GANs could be leveraged to generate synthetic fundus images, thereby supplementing the dataset and potentially boosting model performance. The project could also benefit from further investigation into transfer learning and pre-training. Staying abreast of the latest pre-trained models and fine-tuning strategies would allow researchers to harness the most up-to-date developments in deep learning. Moreover, there is a growing need for interpretable AI models, particularly in the medical domain, where trust and explainability are paramount. Developing models that provide insights into the decision-making process can enhance the acceptance of AI in clinical practice. Clinical validation and deployment are essential steps for translating the research into practical healthcare applications. A crucial next phase is collaborating with medical professionals to validate the models on real-world clinical data and integrate them into healthcare systems. Another intriguing avenue is the assessment of disease severity. Expanding the project to encompass not only disease classification but also the evaluation of disease severity can be vital for treatment planning and monitoring the progression of eye diseases.

The development of real-time detection and monitoring systems should not be overlooked. Such systems could analyse fundus images on the fly, making them suitable for telemedicine and remote healthcare applications. Ethical and privacy considerations must always be a priority. Researchers should include addressing the ethical and privacy aspects of handling medical data, ensuring compliance with data protection regulations, and preserving patient privacy. Furthermore, researchers should aim to collaborate with healthcare institutions to access larger and more diverse datasets. A more extensive dataset can facilitate the training of models that generalise well to a broader range of patients and demographic groups.

## Data Availability

The Ocular Disease Recognition (ODIR) ophthalmic database is available at the following Kaggle link: Ocular Disease Recognition (kaggle.com). The Proposed model code can be shared upon reasonable request to the author, dharunashok113@gmail.com.
